# Ultrasound Imaging of Mouse Fetal Intracranial Hemorrhage Due to Ischemia/Reperfusion

**DOI:** 10.3389/fphys.2017.00340

**Published:** 2017-05-24

**Authors:** Kenichi Funamoto, Takuya Ito, Kiyoe Funamoto, Clarissa L. Velayo, Yoshitaka Kimura

**Affiliations:** ^1^Frontier Research Institute for Interdisciplinary Sciences, Tohoku UniversitySendai, Japan; ^2^Graduate School of Medicine, Tohoku UniversitySendai, Japan; ^3^College of Medicine, University of the PhilippinesManila, Philippines

**Keywords:** antenatal brain injury, fetal intracranial hemorrhage, ischemia/reperfusion, intrauterine growth restriction, cerebrovascular autoregulation, ultrasound imaging

## Abstract

Despite vast improvement in perinatal care during the 30 years, the incidence rate of neonatal encephalopathy remains unchanged without any further Progress towards preventive strategies for the clinical impasse. Antenatal brain injury including fetal intracranial hemorrhage caused by ischemia/reperfusion is known as one of the primary triggers of neonatal injury. However, the mechanisms of antenatal brain injury are poorly understood unless better predictive models of the disease are developed. Here we show a mouse model for fetal intracranial hemorrhage *in vivo* developed to investigate the actual timing of hypoxia-ischemic events and their related mechanisms of injury. Intrauterine growth restriction mouse fetuses were exposed to ischemia/reperfusion cycles by occluding and opening the uterine and ovarian arteries in the mother. The presence and timing of fetal intracranial hemorrhage caused by the ischemia/reperfusion were measured with histological observation and ultrasound imaging. Protein-restricted diet increased the risk of fetal intracranial hemorrhage. The monitoring of fetal brains by ultrasound B-mode imaging clarified that cerebral hemorrhage in the fetal brain occurred after the second ischemic period. Three-dimensional ultrasound power Doppler imaging visualized the disappearance of main blood flows in the fetal brain. These indicate a breakdown of cerebrovascular autoregulation which causes the fetal intracranial hemorrhage. This study supports the fact that the ischemia/reperfusion triggers cerebral hemorrhage in the fetal brain. The present method enables us to noninvasively create the cerebral hemorrhage in a fetus without directly touching the body but with repeated occlusion and opening of the uterine and ovarian arteries in the mother.

## Introduction

Intrapartum asphyxia is a major cause of acute antenatal ischemic stroke and later irreversible neonatal encephalopathy (Cowan et al., 2003). Khatri et al. previously elucidated the pathophysiology for this type of brain injury as a double cascade of events involving ischemia and reperfusion leading to the disruption of the blood brain barrier and eventually, hemorrhagic cerebral transformation (Khatri et al., 2012). Known maternal antenatal risk factors for ischemia/reperfusion injury include primiparity, infertility, infection, pre-eclampsia, gestational diabetes, smoking in pregnancy, and maternal nutrition (Nelson and Lynch, 2004; Wu et al., 2004; Lee et al., 2005; Darmency-Stamboul et al., 2012).

Our recent prenatal programming studies centered on maternal protein restriction illustrate the inherent susceptibility of fetuses and neonates to this type of neural compromise due to intrauterine growth restriction (Velayo et al., 2010, 2014; Dong et al., 2014, 2015). Molecular evidence show both prenatal and postnatal adaptive responses to protein-restricted diets rendering offspring vulnerable to further damage. These findings reinforce the widely-accepted statute that maternal nutrition in pregnancy is critical in early fetal brain development.

Currently, the exact mechanisms by which intrapartum hypoxic-ischemic events lead to acute antenatal ischemic stroke have not been fully elucidated due to the need for time-dependent observation. In this study, we adapted our intrauterine growth restriction mouse model for exposure to ischemia/reperfusion injury to evaluate the presence and timing of fetal intracranial hemorrhage. The ischemia/reperfusion cycles were generated for a mouse fetus by occluding and opening the uterine and ovarian arteries in the mother. Before and during the treatments, time-lapse ultrasound imaging of the fetal brain was performed. In addition, hemorrhage in the fetal brain was determined by histological observation. Our results indicate that protein-restricted diet increases the risk of fetal intracranial hemorrhage caused by ischemia/reperfusion, due to a breakdown in cerebrovascular autoregulation, showing the hemorrhage likely occurs after the second ischemic period.

## Materials and methods

### Experimental animals

This study was carried out in accordance with the recommendations of Regulations for Animal Experiments and Related Activities at Tohoku University, Center for Laboratory Animal Research, Tohoku University. The protocol was approved by the Center for Laboratory Animal Research, Tohoku University. Virgin female C57BL/6N mice (CLEA Japan, Inc., Tokyo, Japan) about 5 weeks old were maintained under controlled lighting (12-h light cycle) and temperature (24°C). These were divided into two diet groups: Normal (N) and Low Protein (LP) by feeding them AIN-93G and modified AIN-93G (Oriental Yeast Co., Ltd., Tokyo, Japan), respectively. They were allowed free access to food and water *ad libitum*. The LP diet consisted of only 48% of the protein and 94% of the calorie contents of the N diet as suggested in previous studies (Reeves et al., 1993; Ito et al., 2011a,b). After a 2-week acclimatization period, the female mice were time mated and inspected for vaginal plugs the following morning. Plug-positive females were then transferred to single cages and fed *ad libitum*. Food consumption and body weight were recorded every day. There was a total of 25 pregnant mice per diet group.

### Ischemia/reperfusion procedure

**Figure 1:**
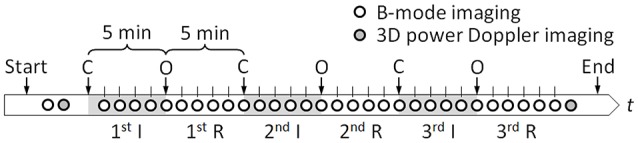
**Time-sequence of the experiment**. Ischemia (I) and reperfusion (R) conditions were created by occluding (C) and opening (O) maternal uterine and ovarian arteries every 5 min. Each circle indicates a timing at which ultrasound B-mode imaging or 3D power Doppler imaging was performed.

We modified Magal's method (Magal et al., 1988) as follows: on day 17.5 of gestation, about 1–2 days before birth, pregnant mice were anesthetized by subcutaneous injection with Ketamine (Ketalar intramuscular 500 mg, Daiichi Sankyo Propharma Co., Ltd., Tokyo, Japan; 10 mg/kg) and Xylazine (Selactar, Bayer Yakuhin, Ltd., Osaka, Japan; 5 mg/kg). Maintenance of anesthesia was achieved using inhalational 0.5% isoflurane at a 200 ml/min air flow rate (Forane, Abbott Japan Co., Ltd., Tokyo, Japan). Each pregnant mouse was placed on a 38°C temperature controlled stage. Maternal abdomen was depilated with commercial hair removal cream (Veet, Reckitt Benckiser Group plc, Slough, England, UK), and this was followed by an abdominal midline incision. The maternal uterine horns containing 2–8 fetuses were exposed, and warmed with ultrasound gel (Parker Laboratories, Inc., Fairfield, NJ, USA) which also enabled ultrasound transmission described later. One fetus was chosen for ischemia/reperfusion treatment. Interval occlusion and opening of maternal uterine and ovarian arteries every 5 min induced ischemia and reperfusion (Figure 1). The ischemia/reperfusion sequence was repeated three times and the whole process lasted a total of 30 min. The number of fetuses treated in each diet group was *n* = 25.

### Ultrasound imaging

Using a Vevo 2100 high-frequency, high-resolution ultrasound device equipped with a linear array transducer MS-550D (VisualSonics, Inc., Toronto, Canada), ultrasound B-mode imaging of a cross section of a fetus brain was performed before and all throughout ischemia/reperfusion intervals (Figure 1). The central frequency of the ultrasound was 40 MHz resulting in the axial resolution of about 40 μm. The field of view was 7 mm in width by 10 mm in depth, and three different focal depths, 4, 6, and 8 mm, were set, simultaneously. One hundred B-mode images were stored at 50 frame/s, wherein each captured cineloop duration was 2 s. Moreover, before and after the ischemia/reperfusion treatment, three-dimensional (3D) ultrasound power Doppler imaging was performed to visualize concomitant blood flow in the area. The scanning length for the 3D imaging was set as 4.98 mm at the interval of 0.102 mm.

### Histology

To evaluate the presence or absence of intracranial hemorrhage after ischemia/reperfusion treatments, the fetuses were delivered and whole brain samples were collected. Brain tissues were supercooled in dry ice for 30 min and stored at −80°C. These were subsequently mounted using an optimal cutting temperature (OCT) compound (Tissue-Tek 4583, Sakura Finetek Japan Co., Ltd., Tokyo, Japan) and cut on a cryostat (Leica Cryostat CM3050 S, Leica Microsystems GmbH, Wetzlar, Germany) to obtain cross sections of 8 μm thickness. Images of brain sections corresponding to ultrasound B-mode images were captured using Leica CTR 5000 and DM 5000B microscope system (Leica Microsystems GmbH, Wetzlar, Germany).

### Image analysis

Changes of blood flow in fetal brains were quantified based on image intensity of ultrasound B-mode images. First, in order to suppress noise in the B-mode images, 100 images obtained at each measurement time point (see Figure 1) were averaged, with adjusting for image shifting due to maternal breathing and heartbeat as well as fetal movement. A customized image analysis program calculated the cross-correlation function between the first image and each subsequent image in the measurement period. Each image was shifted so as to maximize the function, and the time-averaged B-mode image at each time point was then created by averaging image intensities at each pixel. Subsequently, the summation of image intensity, *I*, in a circular region of interest (ROI) with 15-pixel diameters of 0.35 mm was obtained with a separate image-processor (ImageJ, NIH, Bethesda, MD, USA). The ROIs were set at four different locations: cingulate cortex, area 2 (Cg2), basal forebrain (BF), and bilateral caudate putamen (Cpu). Finally, for each ROI, the summation of image intensities, *I*, was normalized with that before the treatment, *I*_0_, and the variation of the relative value of *I*/*I*_0_ was evaluated. A total of five N fetal brains without hemorrhage and five LP fetal brains with hemorrhage, all previously confirmed by above-mentioned histology, were analyzed.

### Statistical analysis

The number of hemorrhage-positive fetuses on histology for each diet treatment group were counted, and the difference between the two groups were evaluated by Fisher's exact test. The differences in intensity of ultrasound B-mode images between the groups were analyzed using a *t*-test. Statistical significance for each analysis was set at *p* < 0.05.

## Results

### Histological findings

**Figure 2:**
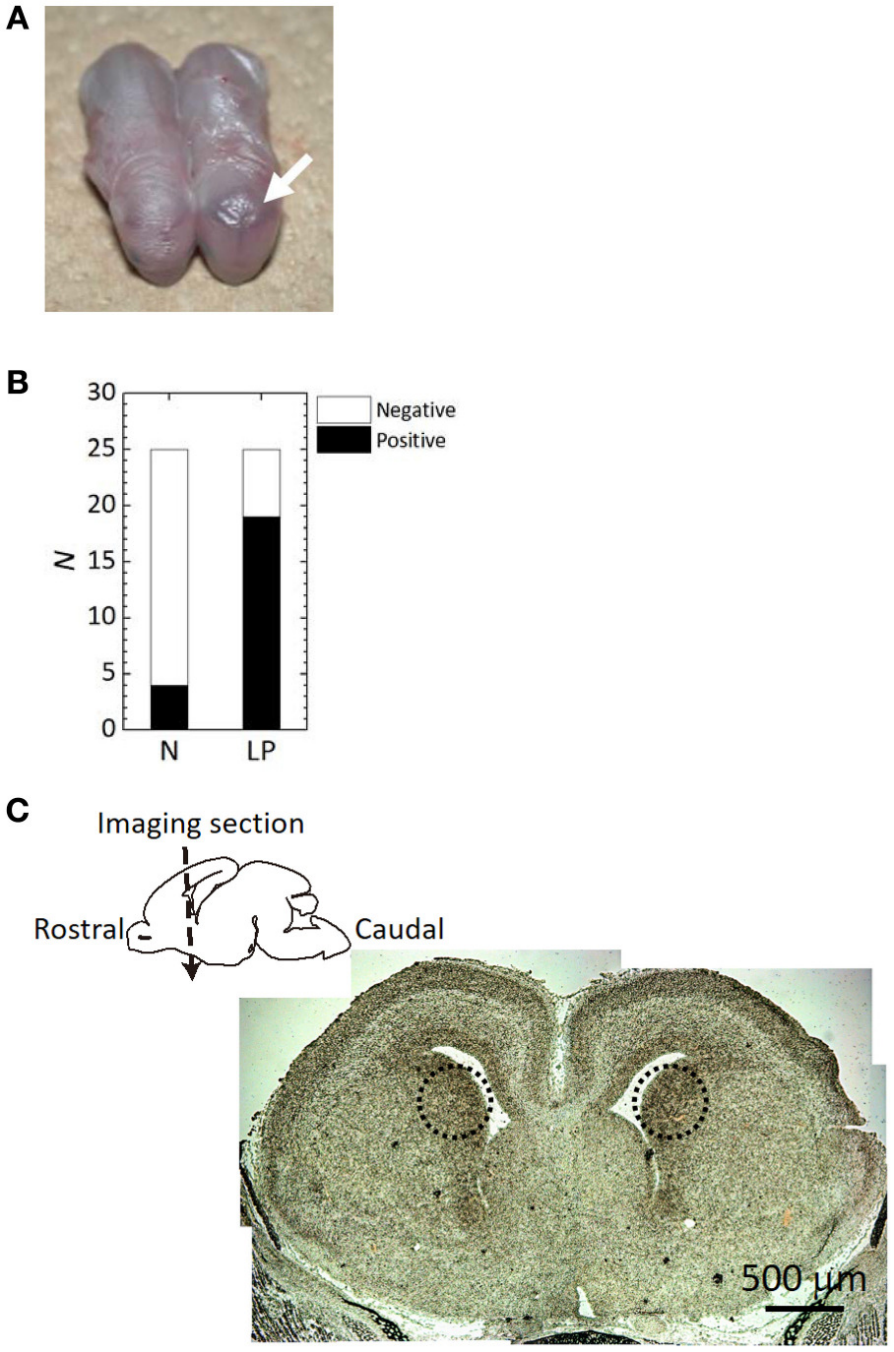
**Histological analysis results after the ischemia/reperfusion treatments. (A)** A snapshot of fetuses with or without intracranial hemorrhage (see an arrow) after the delivery. **(B)** The frequency of hemorrhage occurrence determined by craniotomy of the fetuses in Normal (N) and Low Protein (LP) diet groups. There was a significant difference between the groups (*p* < 0.05). **(C)** Microscopic imaging section of an LP fetal brain on a imaging section. Dotted circles in the microscope image indicate the hemorrhage sites.

A snapshot of fetuses with or without intracranial hemorrhage after ischemia/reperfusion treatments is shown in Figure 2A. A dark region, indicated by a white arrow, in the head of the right-hand side fetus indicates a hemorrhage. The number of fetuses being positive/negative for intracranial hemorrhage determined by histological observation in each group is summarized in Figure 2B. LP fetuses had a 5-fold higher frequency of intracranial hemorrhage than N fetuses; intracranial hemorrhage was noted in 16% and 76% of N and LP groups, respectively. Microscopic observation revealed that bleeding-prone areas were mostly located in the outer peripheral potion of the lateral ventricles, namely Cpu, as observed in the dotted circles in Figure 2C.

### Ultrasonographic findings

**Figure 3:**
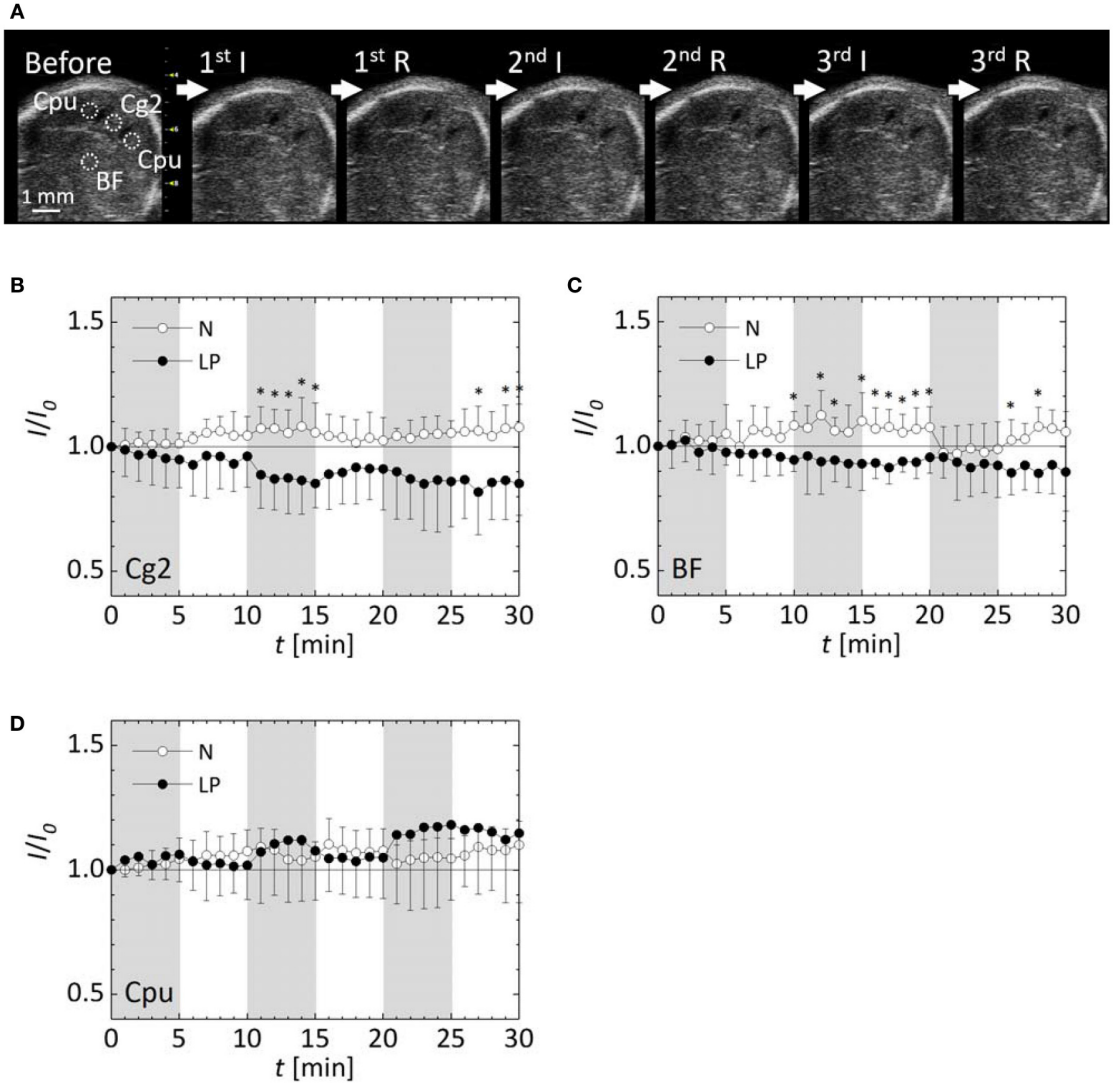
**Observation results by ultrasound B-mode imaging. (A)** A sequential ultrasound B-mode image of a cross section of an LP fetal brain at each phase of the ischemia (I) and reperfusion (R). Dotted circles represent ROIs for quantification of the image intensity. Variations of relative intensities in ROIs in normal (N) and low protein (LP) fetuses: **(B)** the cingulate cortex, area 2 (Cg2), **(C)** the basal forebrain (BF), and **(D)** the bilateral caudate putamen (Cpu). Five fetuses were analyzed for each case. Shaded time intervals are ischemic period, and the other ones are reperfusion period. Error-bars represent standard deviation. ^*^p < 0.05.

Sequential ultrasound B-mode images of the same cross section in Figure 2C were successfully obtained during the ischemia/reperfusion treatments as shown in Figure 3A. This achievement enabled us to quantify changes in blood flow by intensity analysis of the sequential images described later with Figures 3B–D. At the Cpu beside the lateral ventricles where intracranial hemorrhage observed in the microscopic image, increases of the intensity were recognized in the sequential ultrasound B-mode image.

**Figure 4:**
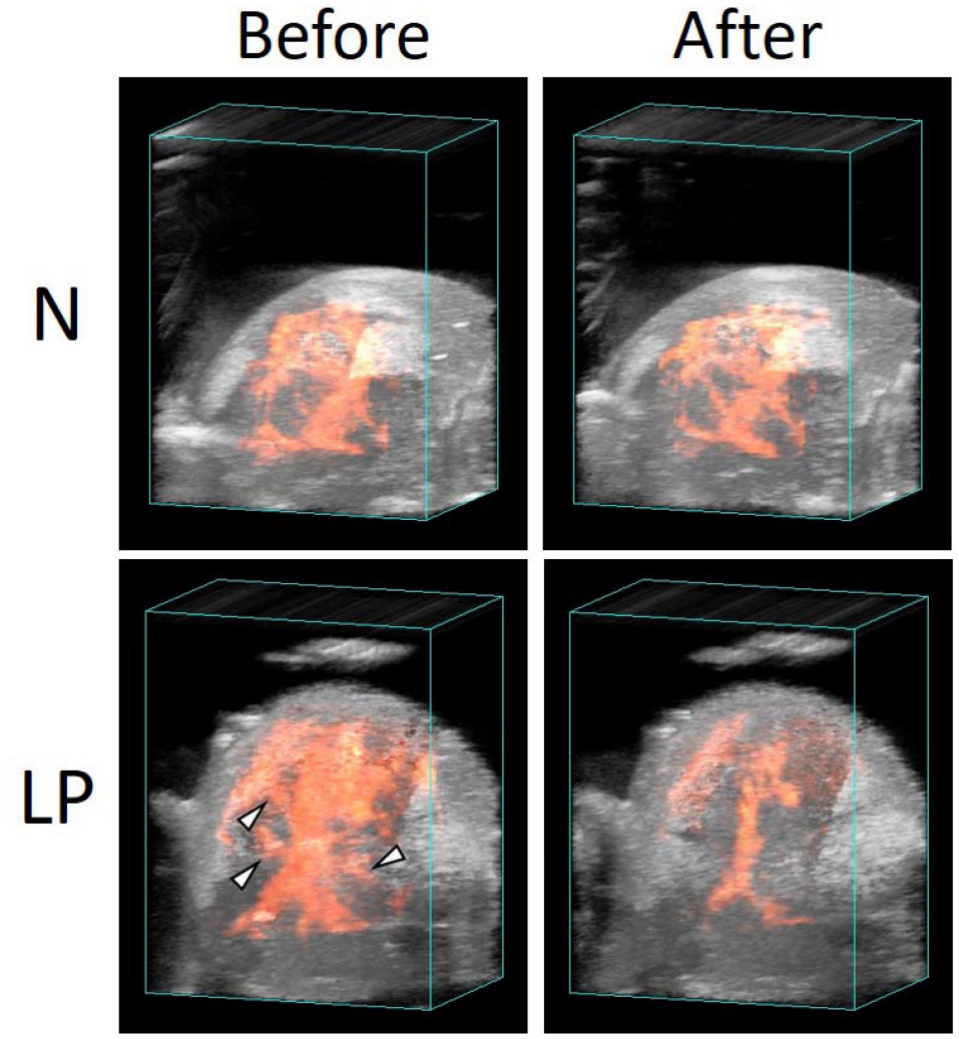
**Vascular structures in normal (N) and low protein (LP) fetal brains before and after the ischemia/reperfusion treatments, reconstructed by 3D power Doppler imaging**.

Blood flow in N and LP fetal brains before and after the ischemia/reperfusion treatments are demonstrated in 3D ultrasound power Doppler images of Figure 4. No significant change was observed in the N fetal brain before and after the treatment. On the other hand, several main blood flows disappeared in the LP fetus as indicated by triangles in Figure 4.

### Intensity analysis findings

Changes in blood flow within the fetal brain were evaluated by intensity quantification of ultrasound B-mode images with each five N and LP mice. Sequential ultrasound B-mode images of five N fetal brains without hemorrhage and five LP fetal ones with hemorrhage, confirmed by histological observation using microscopy, were analyzed. The variations of the relative intensities in ultrasound B-mode images in the four ROIs are shown in Figures 3B–D. Note that the results of Cpu were obtained by combining 10 data sets of bilateral Cpu in five fetuses. Image intensities in all the ROIs of N fetal brains without hemorrhage were slightly increased with fluctuations due to ischemia/reperfusion treatments. In contrast, the image intensities of LP fetal brains gradually decreased in the Cg2 and BF ROIs, but increased in Cpu ROI, showing a large standard deviation. The relative intensities in the Cg2 and BF ROIs of LP fetal brains were significantly lower than those in N fetal brains at some time points after the second ischemic period. No significant difference was detected between the relative intensities in the Cpu ROI between N and LP fetuses, but the fluctuation of relative intensity in the Cpu ROI during the ischemic/reperfusion treatments were opposite between the two groups.

## Discussion

The present study provides further evidence that ischemia/reperfusion events lead to the evolution of antenatal stroke. Moreover, it supports the assertion that the frequency of fetal cerebral hemorrhage increases in conditions of maternal-fetal undernutrition. It is known that fetal undernutrition causes diseases or disorders after birth or in adulthood (Woodall et al., 1996). The maternal/fetal low-protein condition also contributes to fetal cerebral hemorrhage, showing that the frequency of fetal cerebral hemorrhage was statistically increased as shown in Figure 2B. Most bleeding-prone areas were the outer peripheral potion of the lateral ventricles of Cpu as displayed in Figure 2C. This tendency of fetal intracranial hemorrhage corresponds to the reference (Volpe, 2001), which reviewed an increased likelihood of periventricular leukomalacia in the presence of intraventricular hemorrhage.

Hypoxic condition was created by occluding the uterine and ovarian arteries and holding blood at the placenta. It is reported that brain cells can endure 15-min oxygen deficiency, and the present method of three times 5-min occlusion of the maternal arteries corresponds to the circumstance. Former studies utilized an animal model of rabbit for cerebral hemorrhage by repeatedly occluding and opening the carotid artery. In the animal model, blood supply to the brain was physically interrupted, and was impractical to apply to investigation of fetal intracranial hemorrhage. The present method enables us to noninvasively induce cerebral hemorrhage in a fetus through maternal arterial occlusion and directly observe the physiologic phenomenon of fetal brain sparing though real-time ultrasonography.

The variations of the relative intensity of ultrasound B-mode images represented different tendencies between the ROIs and nutrient conditions. In all ROIs, the blood flow in the N fetus retained or rather slightly increased during ischemia period. This implies that the N fetus has cerebrovascular autoregulation to maintain a constant blood supply to the fetal brain during the ischemia/reperfusion. In contrast, in both Cg2 and BF ROIs, cerebral blood flow in the LP fetuses decreased in the ischemia period and slightly returned in the following reperfusion period (Figures 3B,C). Whereas, the blood flow in Cpu of LP fetuses increased and became unstable with a relatively large deviation after the second ischemic period (Figure 3D). Interestingly, fluctuations of relative intensity in the Cpu ROI were opposite between N and LP fetuses. In 3D ultrasound power Doppler imaging of an LP fetal brain (Figure 4), decreased blood flow in a large tributary of the anterior cerebral artery was clearly recognized (Dorr et al., 2007). From all these observations, it may be concluded that the LP fetal brain fails to maintain blood flow during conditions of ischemia/reperfusion due to autoregulatory malfunction.

This study revealed “where” and “when” the intracranial hemorrhage occurs. However, “why” it occurs should be further investigated regarding mechanical weakness of a fetal intracranial vasculature and cell death. Decrease of blood flow in LP fetus observed in this study can be caused by lessened heart rate and decreased cardiac output. Further investigation would be performed by monitoring both maternal and fetal heart conditions with electrocardiography. Prevention method for fetal intracranial hemorrhage is also future work.

## Conclusion

The presence and timing of fetal intracranial hemorrhage caused by ischemia/reperfusion injury were evaluated using an intrauterine growth restriction mouse model. Histological analysis and ultrasound imaging were performed on mouse fetuses undergoing ischemia/reperfusion cycles which were generated by occluding and opening the uterine and ovarian arteries in the mother. Protein-restricted diets increase the risk of fetal intracranial hemorrhage due to a breakdown in cerebrovascular autoregulation.

## Author contributions

TI and YK designed the study; all authors substantially contributed to the experiments; TI and KiF took care of mice with specialized diet; TI performed surgical treatments, KeF and KiF measured fetal brains with ultrasound equipment with supervision of YK; TI performed histological observation; KeF analyzed ultrasound B-mode images; and KeF, TI, and CV wrote the manuscript with contribution of all authors.

### Conflict of interest statement

The authors declare that the research was conducted in the absence of any commercial or financial relationships that could be construed as a potential conflict of interest.
